# Supplementary material for: The peptidase DA1 cleaves and destabilizes WUSCHEL to control shoot apical meristem size
Source: Nat Commun. 2024 May 31;15:4627. doi: 10.1038/s41467-024-48361-7 (PMC11143343; doi:10.1038/s41467-024-48361-7)

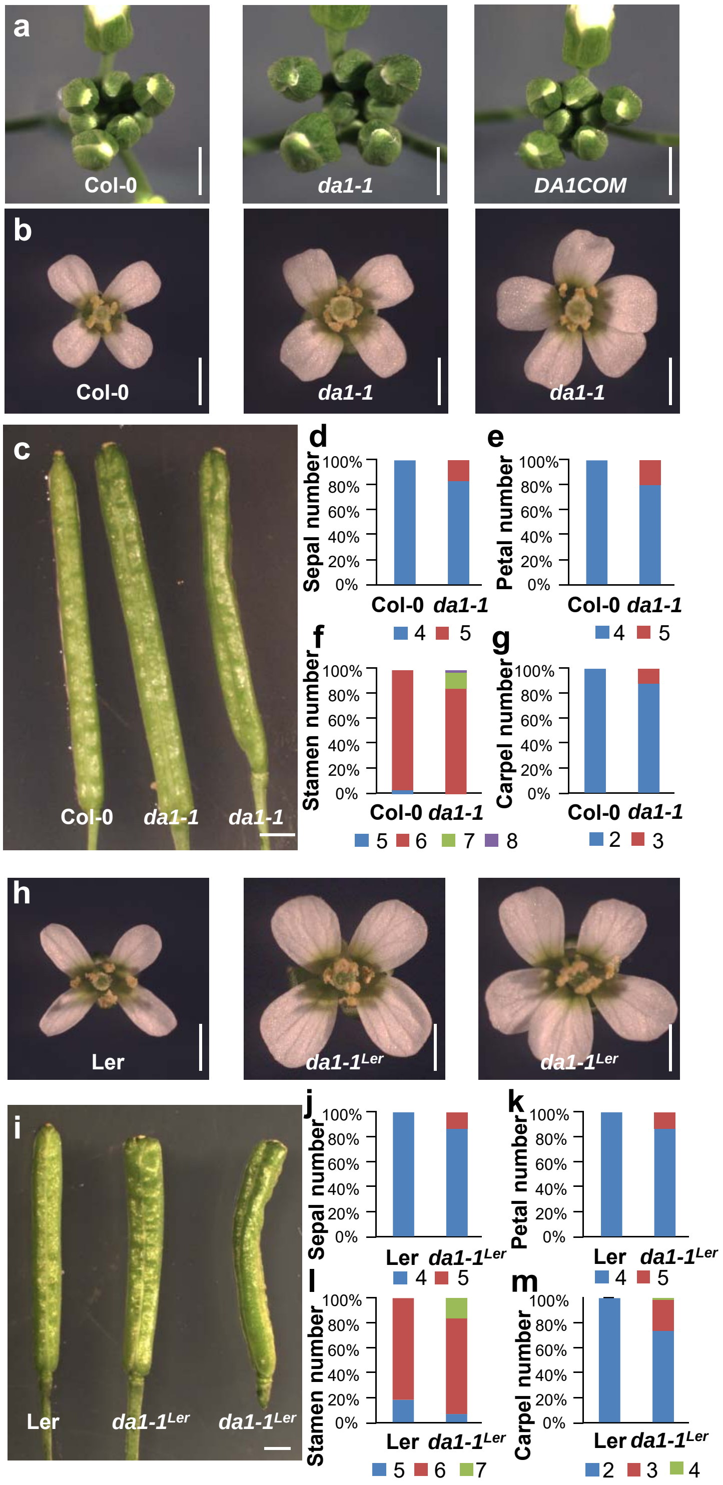

Supplementary Fig. 1. The *da1-1* and *da1-1*^{Ler} mutants show the increased number of floral organs. a The inflorescences of Col-0, *da1-1* and *DA1COM*. The *da1-1* forms large inflorescences. *DA1COM* represents that *da1-1* mutant was transformed with the *DA1* genomic DNA. **b** The flowers of Col-0 and *da1-1*. The *da1-1* mutant contains 4 or 5 petals in one flower. **c** The siliques of Col-0 and *da1-1*. The *da1-1* mutant contains 2 or 3 carpels in one silique. **d-g** The number of sepals, petals, stamen and carpels in Col-0 and *da1-1*(n=60). **h** The flowers of Ler and *da1-1*^{Ler}. The *da1-1*^{Ler} mutant contains 4 or 5 petals in one flower. **i** The silique phenotype of Ler and *da1-1*^{Ler}. *da1-1* mutants contain 2 or 3 carpels in one silique. **j-m** The numbers of sepals, petals, stamen and carpels in Ler and *da1-1*^{Ler}(n=60). Scale bars, 1mm (**a, b, c, h, i**). The experiments were repeated independently at least twice with similar results.

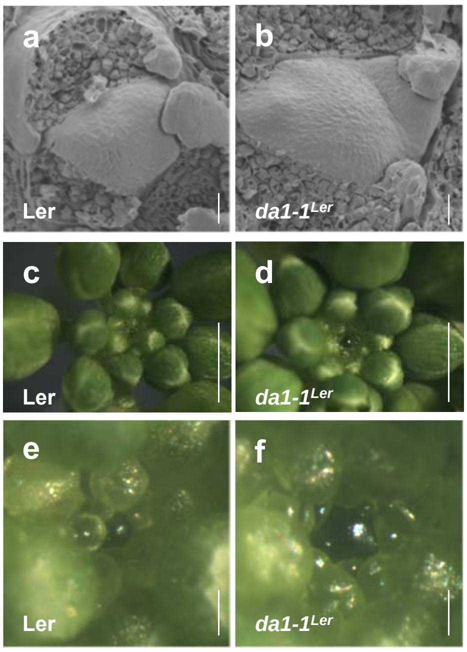

Supplementary Figure. 2. *da1-1^{Ler}* displays large SAM and IM. a, b SEM images for the shoot apical meristem (SAM) of Ler (n=21) and *da1-1^{Ler}* (n=34). The *da1-1^{Ler}* had larger SAM than Ler. Plants were grown for 6 days in long-day conditions. **c-f** The inflorescence meristem (IM) of Ler (n=17) and *da1-1^{Ler}* (n=16) . The *da1-1^{Ler}* had larger IM than Ler. Scale bars, 20μm (**a, b**), 1mm(**c, d**) 100μm (**e, f**). The experiments were repeated independently at least twice with similar results.

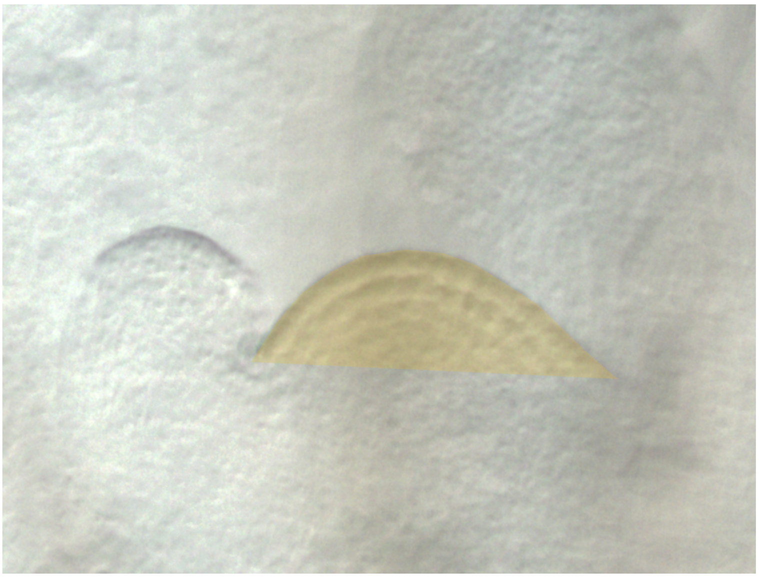

Supplementary Figure. 3. The measurement for the SAM area.

Yellow color shows the area of the SAM.

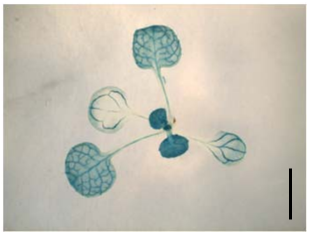

Supplementary Figure. 4. GUS staining of *pDA1:GUS* plants. Images of *pDA1:GUS* plants. Plants were grown for 16 days in long-day conditions and then stained by GUS buffer. Scale bars, 3mm。

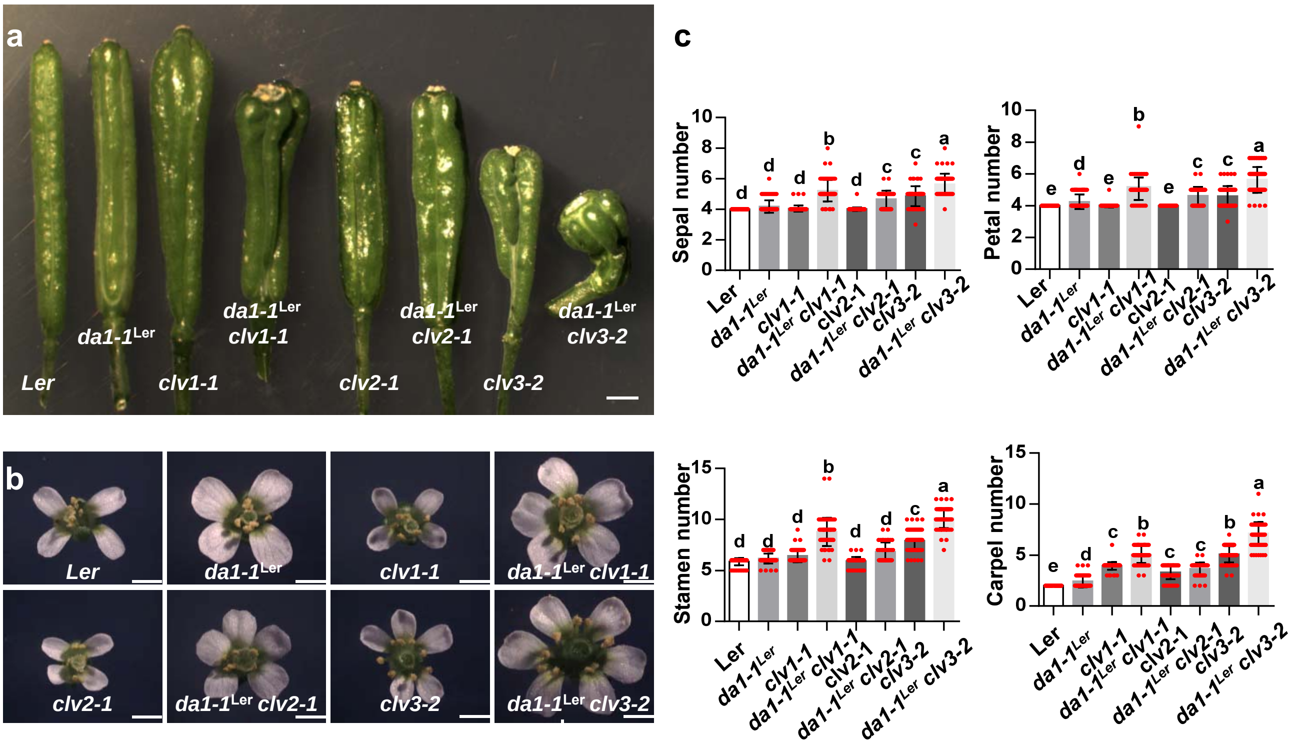

Supplementary Figure. 5. The *da1-1*^{Ler} enhances the floral organ numbers of *clv* mutants. a The silique phenotypes of Ler, *da1-1*^{Ler}, *clv1-1*, *da1-1*^{Ler} *clv1-1, clv2-1*, *da1-1*^{Ler} *clv2-1, clv3-2* and *da1-1*^{Ler} *clv3-2*. **b** The flower phenotypes of Ler, *da1-1*^{Ler}, *clv1-1*, *da1-1*^{Ler} *clv1-1, clv2-1*, *da1-1*^{Ler} *clv2-1, clv3-2* and *da1-1*^{Ler} *clv3-2*. **c** Statistics of the floral organ number in Ler, *da1-1*^{Ler}, *clv1-1*, *da1-1*^{Ler} *clv1-1, clv2-1*, *da1-1*^{Ler} *clv2-1* , *clv3-2* and *da1-1*^{Ler} *clv3-2*(n = 49). Data are presented as mean values ± s.e.m. One-way ANOVA with Tukey's multiple comparison test was used for statistical analyses($P < 0.05$). Scale bars, 1mm (**a, b**). The experiments were repeated independently at least twice with similar results.

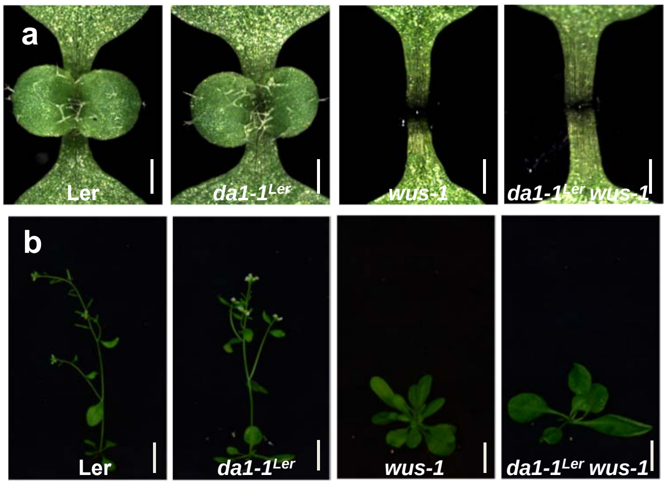

Supplementary Figure. 6. The *wus-1* is epistatic to *da1-1^{Ler}*. **a** Ler, *da1-1^{Ler}*, *wus-1* and *da1-1^{Ler} wus-1* seedlings. Plants were grown for 6 days in long-day conditions. **b** The 35-day-old Ler, *da1-1^{Ler}*, *wus-1* and *da1-1^{Ler} wus-1* plants. Scale bars, 50μm(**a**), 1.5 cm(**b**).

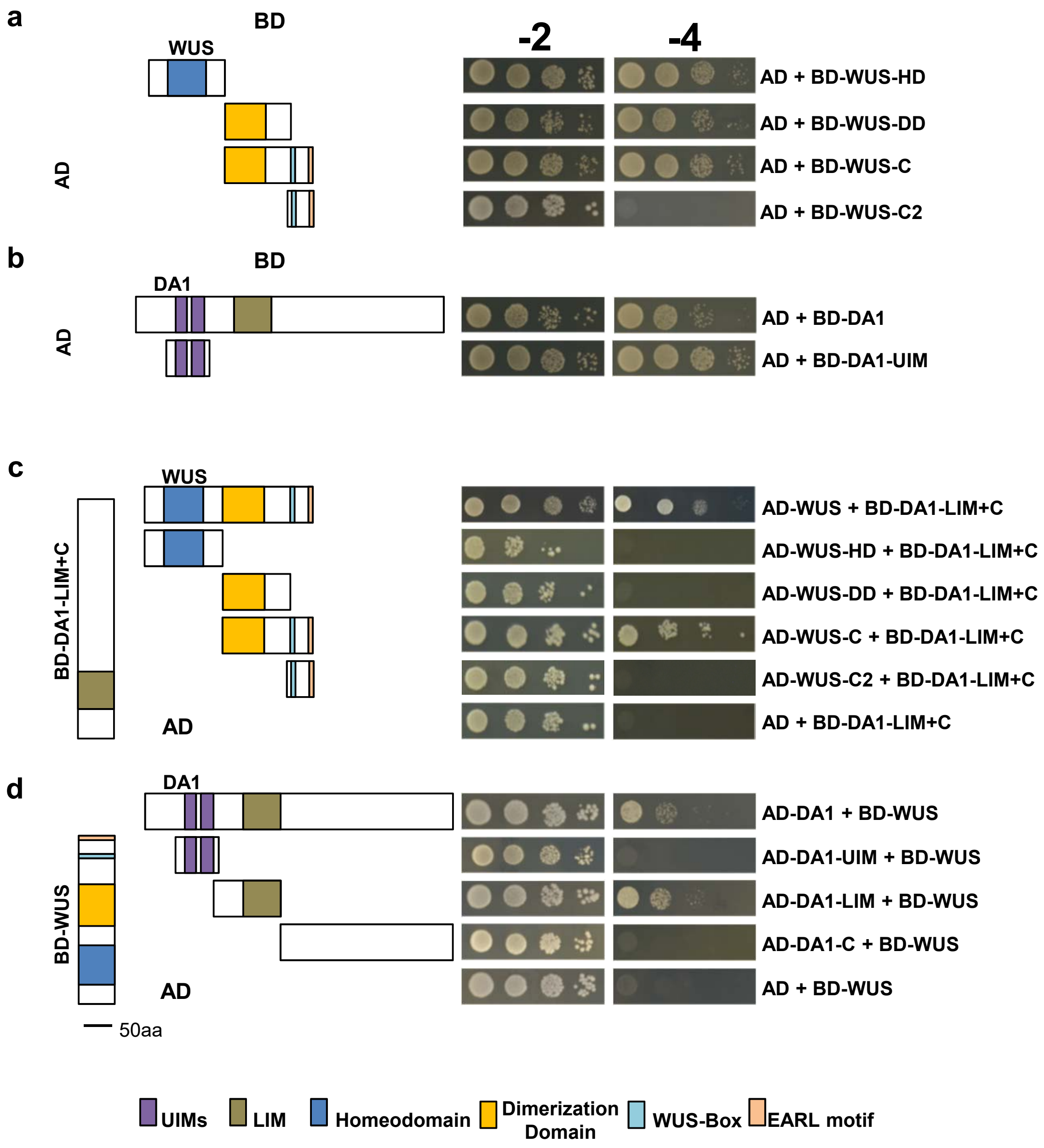

Supplementary Figure. 7. DA1 physically interacts with WUS. The indicated construct pairs were co-transformed into yeast strain Y2H Gold (Clontech). Interactions between bait and prey were examined on the control media DDO (SD/-Leu/-Trp) and selective media QDO (SD/-Ade/-His/-Leu/-Trp). **a** The HD domains (Homeodomain), DD domains (Dimerization Domain) and C terminal region (Dimerization Domain,WUS-Box and EARL motif) fused to BD autoactivated the reporter gene. **b** The full length DA1 or the UIM domains fused to BD autoactivated the reporter gene. **c** BD-DA1-LIM+C interacted with AD-WUS C terminal region (the dimerization domain, WUS-box and EARL motif). **d** BD-WUS interacted with full length DA1 and LIM domains fused to AD.

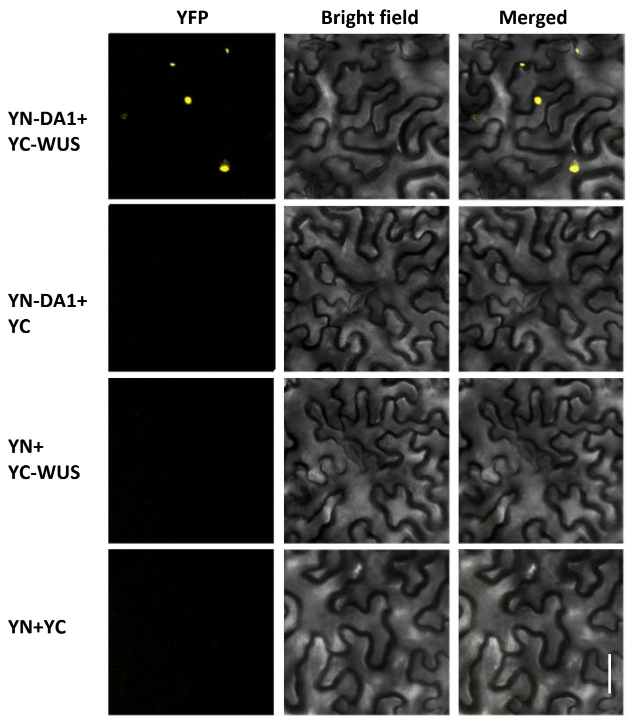

Supplementary Figure. 8. DA1 associates with WUS in nuclei.
Bimolecular fluorescence complementation (BiFC) assays in *N. bentheamiana* leaves showed that DA1 associates with WUS in nuclei. nYFP, N-terminal portion of YFP; cYFP, C-terminal portion of YFP. Scale bars, 50 μm.). The experiments were repeated independently at least twice with similar results.

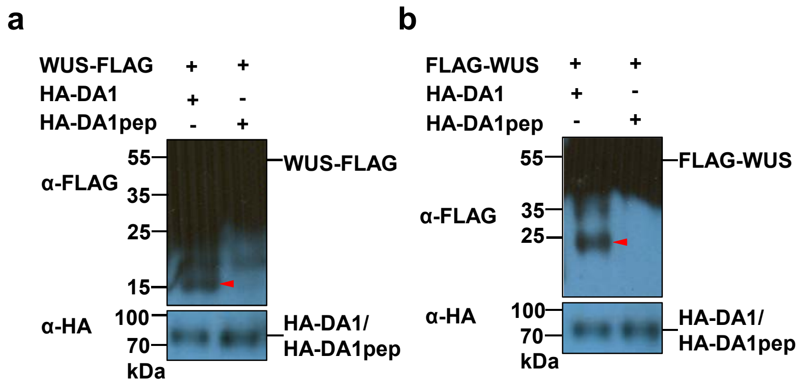

Supplementary Figure. 9. DA1 cleaves WUS. a, **b** Arabidopsis *da1-ko1 dar1-1* mesophyll protoplasts were cotransfected with plasmids expressing WUS-FLAG or FLAG-WUS with HA-DA1 and HA-DA1pep, respectively. The specific cleavage products of WUS-FLAG and FLAG-WUS were indicated by red arrowheads.

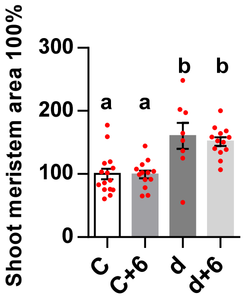

Supplementary Figure. 10. The size of shoot meristerm was not affected by 6-BA treatment for 8h. The average area of SAMs of *pWUS:WUS-GFP* (C) (n=15), *pWUS:WUS-GFP* + 6-BA (C + 6) (n=13), *pWUS:WUS-GFP; da1-1* (d) (n=8) and *pWUS:WUS-GFP; da1-1* + 6-BA (d + 6) (n=13) SAMs. Different letters denote significant differences ($P < 0.05$) from a Tukey's multiple comparisons test. Data are mean ± s.e.m. relative to the C value (100%). Plants were grown for 8.67 days on 1/2MS solid medium and transferred to 1/2MS solid medium with 6-BA for 8 hours. The experiments were repeated independently at least twice with similar results.

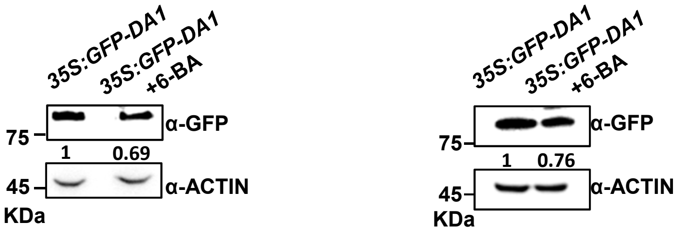

Supplementary Figure. 11. CK modulates the stability of DA1 protein. The DA1 protein accumulates at lower levels in the *35S:GFP-DA1* transgenic plants treated with 6-BA. Total protein extracts were subjected to immunoblot assays using anti-GFP and anti-ACTIN (as loading control) antibodies. (two repeats of Fig. 6a).

Raw data for blots

Fig3b

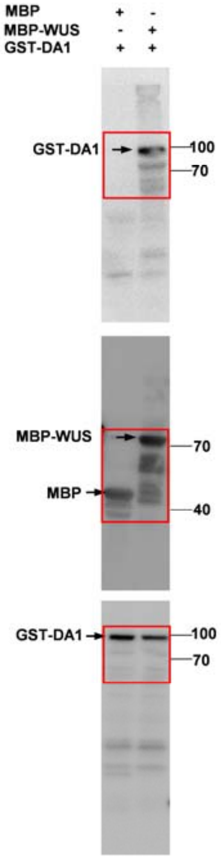

Fig3c

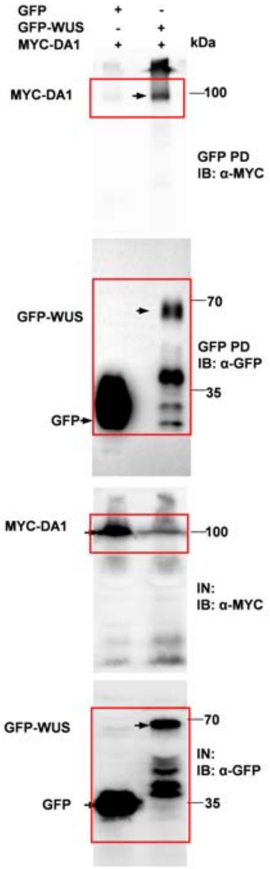

Fig3d

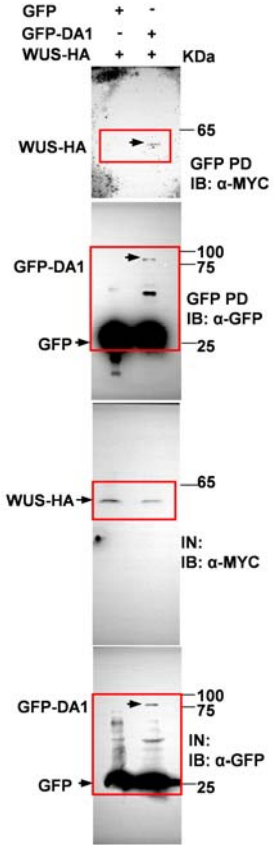

Fig3e

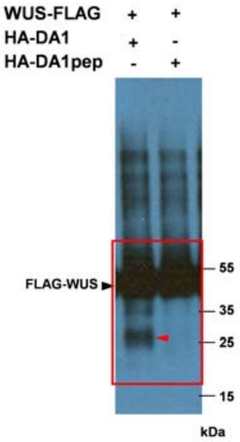

Fig3f

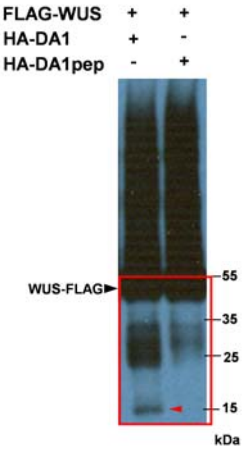

Supplemental Fig. 8.

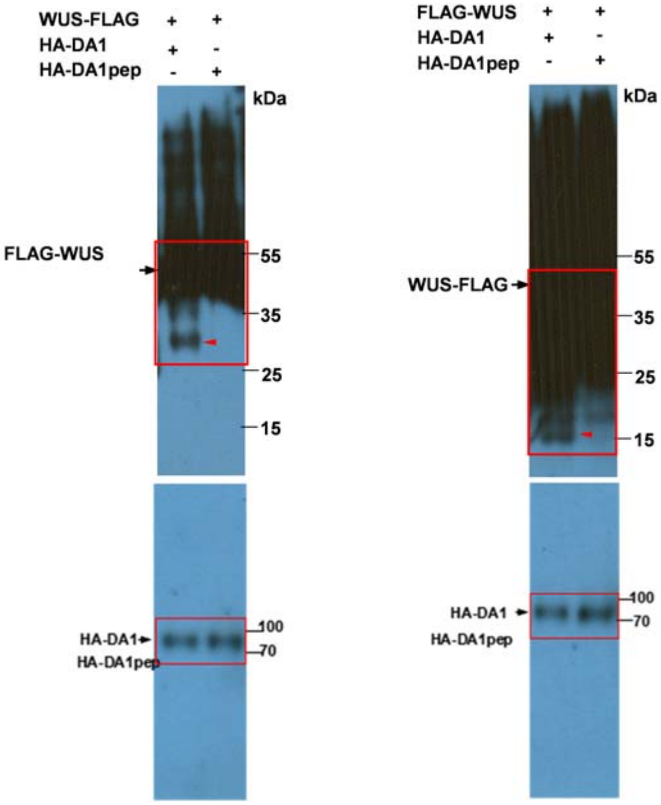

Fig4k

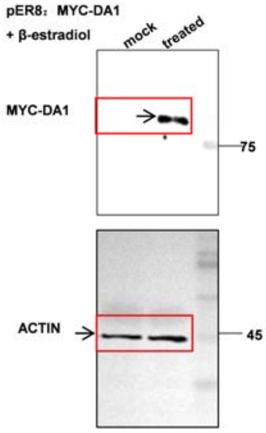

Fig6a

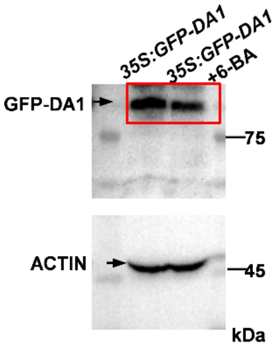

Supplemental Fig. 9.

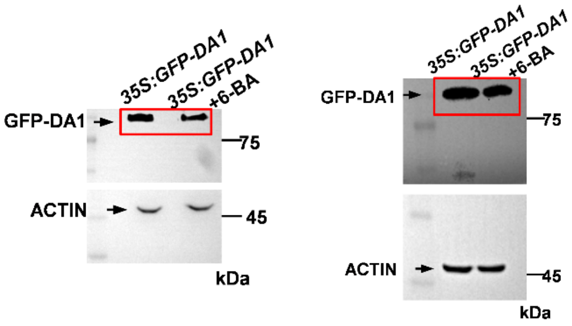

Supplement: Supplementary file 1 — Supplementary Information [file 41467_2024_48361_MOESM1_ESM.pdf]
