# Supplementary material for: The peptidase DA1 cleaves and destabilizes WUSCHEL to control shoot apical meristem size
Source: Nat Commun. 2024 May 31;15:4627. doi: 10.1038/s41467-024-48361-7 (PMC11143343; doi:10.1038/s41467-024-48361-7)
Supplement: Supplementary file 3 — Supplementary Data 1 [file 41467_2024_48361_MOESM3_ESM.docx]

| **Supplementary Data 1 \| List of primers used in this study** | |
| --- | --- |
| **Primers for genotyping mutant lines** | |
| *da1-1* MnlI-CAPS-LP | ACAAGCACTTAACGAAGCCAGAG |
| *da1-1* MnlI-CAPS-RP | GAACAAAATTCAGCAAAACCATC |
| *wus-1* KpnI-dCAPS-LP | ATATGTTTGAAGGGAATTTAAATCATGC |
| *wus-1* KpnI-dCAPS-RP | AATTAATGAATTATAGTTTGGTAGGTA |
| *wus-7* TaqI-CAPS-LP | TCACCAACAGCCGATCAGAT |
| *wus-7* TaqI-CAPS-RP | CCGCCATCATAACCGAGTTG |
| **Primers for qRT-PCR** | |
| APS4-qF | CATCATGCTTCTCGTTATGCTC |
| APS4-qR | CATGTACCCACTCTAGATCCAC |
| SNX2a-qF | GGAGTTGAATTCCCAAACTGTC |
| SNX2a-qR | CTAGATCTATCTGCAAATGCGC |
| TPL-qF | CCAATTAAACAGACCCTGCTTC |
| TPL-qR | CTCAAATCCCAGACTTTGAACG |
| BCAT4-qF | GCAACTGGCAAAAACATTGAAG |
| BCAT4-qR | AATTCCATTACGCAGTTTCGAG |
| ARR5-qF | CACTTCTTCATTAGCATCACCG |
| ARR5-qR | CAGGCATAGAGTAATCCGTCAT |
| ARR7-qF | TGAGGTCATGAGGATGGAGATTC |
| ARR7-qR | CAAGATACTGCAAAGCCCTAGTTC |
| WUS-qF | GCAAGAACGTCTTTTACTGGTT |
| WUS-qR | GATGATAATGATCGTTAGCCGC |
| GFP-qF | taatgggcacaaattttctgtc |
| GFP-qR | tggccatggaacaggtagtt |
| DA1-qF | ACTTTGCCTTGAGTGTTTGG |
| DA1-qR | TGCTTTGATCGCTTTCTTAC |
| **Primers for molecular cloning** | |
| MBP-WUS-F | atcgagggaaggatttcagaattcGAGCCGCCACAGCATCAGCA |
| MBP-WUS-R | ccagtgccaagcttgcctgcagCTAGTTCAGACGTAGCTCAA |
| GFP-WUS-F | catggatgaactatacaaaggcgcgccaGAGCCGCCACAGCATCAGCA |
| GFP-WUS-R | gccgctctagaactagttaattaaCTAGTTCAGACGTAGCTCAA |
| WUS:WUS-HA-F | aaacgacggccagtgccaagcttacgtacggtgtgtgtgtttg |
| WUS:WUS-HA-R | gccgctctagaactagttaattaaCTATACCCATACGACGTCCCAGACTACGCTGTTCAGACGTAGCTCAA |
| WUS-FLAG-PW1211-F | GGGGACAAGTTTGTACAAAAAAGCAGGCTCAATGGAGCCGCCACAGCATCA |
| WUS-FLAG-PW1211-R | GGGGACCACTTTGTACAAGAAAGCTGGGTAGTTCAGACGTAGCTCAA |
| FLAG-WUS-PW1266-F | GGGGACAAGTTTGTACAAAAAAGCAGGCTCAATGGAGCCGCCACAGCATCA |
| FLAG-WUS-PW1266-R | GGGGACCACTTTGTACAAGAAAGCTGGGTACTAGTTCAGACGTAGCTCAA |
| pER8:MYC-DA1-F | CTGAAGCTAGTCGACTCTAGCCTCGAGgctatggagcaaaagctcat |
| pER8:MYC-DA1-R | GAGGCCTGGATCGACTAGTTAATTAATTAAACCGGGAATCTACCGG |
| BD-WUS-F | catatggccatggaggccgaattcATGGAGCCGCCACAGCATCA |
| BD-WUS-R | cggccgctgcaggtcgacggatccCTAGTTCAGACGTAGCTCA |
| MYC-DA1pep-F | acttgaattcggtacccATGGGTTGGTTTAACAAGATC |
| MYC-DA1pep-R | taggctacgtaggatccaTTAAACCGGGAATCTACCGGTC |
| YN-F | agaacacgggggactctagATGGTGAGCAAGGGCGAG |
| YC-F | agaacacgggggactctagATGGCCGACAAGCAGAAG |
| YN-R-DA1 | GATCTTGTTAAACCAACCCATttccataggcatatactc |
| YN-DA1-F | gagtatatgcctatggaaATGGGTTGGTTTAACAAGATC |
| YN-DA1-R | atatccagtcactatggtcgaTTAAACCGGGAATCTACCGGTC |
| YC-R-WUS | CTGATGCTGTGGCGGCTCCATcgcatagtcaggaacatc |
| YC-WUS-F | gatgttcctgactatgcgATGGAGCCGCCACAGCATCAG |
| YC-WUS-R | atatccagtcactatggtcgaCTAGTTCAGACGTAGCTCAAG |
| pWUS-107-F | catgcctgcaggtcgactctagaacgtacggtgtgtgtgtttg |
| pWUS-107-R | gggccccccctcgaggcgcgccagtgtgtttgattcgacttttg |
| pWUS-DA1CDS-107-F | cgaatcaaacacactggcgcgccATGGGTTGGTTTAACAAGAT |
| pWUS-DA1CDS-107-R | cgggccccccctcgaggcgcgccaAACCGGGAATCTACCGGTCA |
| WUS-AD-F1 | CCATGGAGGCCAGTGAATTCATGGAGCCGCCACAGCATCAG |
| WUS-AD-F2 | CCATGGAGGCCAGTGAATTCATGATGCAGAGACCTGCTA |
| WUS-AD-F3 | CCATGGAGGCCAGTGAATTCATGCGACGTACGCTTC |
| WUS-AD-R1 | TTCATCTGCAGCTCGAGCTCGGGAACACCGTGATGATGGT |
| WUS-AD-R2 | TTCATCTGCAGCTCGAGCTCATGTTCCAGATAAGCATCG |
| WUS-AD-R3 | TTCATCTGCAGCTCGAGCTCAACTTCCGATTGGCCATACT |
| WUS-AD-R4 | TTCATCTGCAGCTCGAGCTCCTAGTTCAGACGTAGCTCAA |
| WUS-BD-F1 | GGACCTGCATATGGCCATGGAGATGGAGCCGCCACAGCATCAG |
| WUS-BD-F2 | GGACCTGCATATGGCCATGGAGATGATGCAGAGACCTGCTA |
| WUS-BD-F3 | GGACCTGCATATGGCCATGGAGATGCGACGTACGCTTC |
| WUS-BD-R1 | TAGTTATGCGGCCGCTGCAGGGGGAACACCGTGATGATGGT |
| WUS-BD-R2 | TAGTTATGCGGCCGCTGCAGGATGTTCCAGATAAGCATCG |
| WUS-BD-R3 | TAGTTATGCGGCCGCTGCAGGAACTTCCGATTGGCCATACT |
| WUS-BD-R4 | TAGTTATGCGGCCGCTGCAGGCTAGTTCAGACGTAGCTCAA |
